# The recovery experiences of individuals with schizophrenia in northwestern Ethiopia

**DOI:** 10.3389/fpsyt.2025.1470656

**Published:** 2025-03-03

**Authors:** Selamawit Kassa, Setareh Ghahari, Rosemary Lysaght, Carrie Anne Marshall, Missaye Mulatie Mengstie

**Affiliations:** ^1^ Department of Rehabilitation Science, School of Rehabilitation Therapy, Queen's University, Kingston, ON, Canada; ^2^ School of Occupational Therapy, Western University, London, ON, Canada; ^3^ Department of Psychology, University of Gondar, Gondar, Ethiopia

**Keywords:** schizophrenia, recovery, coping mechanisms, family support, mental health

## Abstract

**Introduction:**

Schizophrenia recovery is perceived as a one-time symptom remission. However, it is a process that may continue throughout an individual's life. Understanding the recovery experience of a person with schizophrenia is an essential component of filling the mental health service gap and enhancing a smooth recovery process. The objective of this study was to explore the recovery experience of individuals with schizophrenia in a developing country.

**Methods:**

To understand these experiences, we used qualitative research. Seventeen participants were purposefully selected from two hospitals in Northwestern Ethiopia. Semi-structured, in-depth interviews were conducted, and data was analyzed using Interpretive Phenomenological Analysis.

**Results:**

Finally, four themes were generated: the first theme was the meaning of recovery. The definition of schizophrenia recovery differs from person to person based on their personal experience. For some, recovery means becoming free of symptoms, and for others, helping their family and being economically independent. The second theme was obstacles to recovery. The obstacles included limited understanding from family members, decreased social engagement, financial dependency, hopelessness, and suicidal thoughts. The third theme was support by family and employers. Participants received support from people around them. Family members mainly provided support by feeding, clothing, giving medications, and taking them to spiritual places. The fourth theme was coping strategies used to manage illness, which included medication adherence, visiting holy water sites and traditional healers, and listening to spiritual lessons.

**Conclusion:**

Generally, participants focused on recovery from illness by emphasizing being free from symptoms to start their regular life. However, some of them were interested in pursuing education and engaging in work. The meaning given for recovery determines the kind of recovery process individuals have. Accepting that schizophrenia is a lifelong disorder, following medical treatments, finding ways to be active in their social life, and engaging in day-to-day activities are crucial components of recovery. In addition, since support from family members is vital, creating awareness creation programs for family members and the community is essential. Finally, psychosocial support and vocational training are important strategies that need to be considered to support recovery.

## Introduction

Schizophrenia recovery has been characterized as a dynamic and individualized journey hinging on a person’s capacity to rediscover and reassert their sense of self (1). *Recovery* is not a destination to be achieved but rather a personal commitment to the active and ongoing process of well-being (2). Generally, there are two schools of thought around recovery: ‘recovery from’ and ‘recovery in’. If the individual or their healthcare provider defines recovery as ‘recovery from’ the illness, symptom relief is emphasized as a means toward the end of an individual’s restored health following the onset of a serious mental illness. In contrast, those who consider recovery as ‘recovery in’ mental illness focus on the means of identifying and developing an individual’s strengths and strategies to reduce the influence of the illness on their well-being and identity; in following these principles, *recovery in* schizophrenia prioritizes symptom management, optimization of existing clinical and rehabilitative practices, and the importance of establishing a meaningful life within one’s community regardless of their disability status (1).

It is important to note that *the recovery experience* is highly context-dependent, influenced by individuals’ uniquely personal experiences. However, no matter the diversity of lived experiences, everyday experiences can often be found at the intersections of individuals’ identities, such as the common realities experienced by persons with schizophrenia. For many, the onset of schizophrenia results in profound loneliness and detachment from society, withdrawal from occupational pursuits such as education, and impediments to gainful employment (3). A study conducted in the United States (US) illustrated the series of states, or phases across one’s lifespan, experienced by individuals living with schizophrenia. Upon diagnosis, a person may feel overwhelmed and distressed due to fear and disbelief. Adjusting to this diagnosis is often emotionally intensive, beginning with a great uncertainty that drives the pursuit of knowledge toward understanding one’s re-envisioned life trajectory, bringing grief in tow. Many individuals and their families particularly struggle with the life-long reality of schizophrenia. In time, these individuals gradually learn how to live in the context of their disability, regaining a sense of self-actualization, -efficacy, and -autonomy through recovery (4). However, recovery is itself marked by confrontations with more *social* symptoms of schizophrenia. Individuals living with schizophrenia commonly experience social and self (i.e., internalized) stigma founded on fears derived from ignorance, a lost sense of autonomy or hope in life, and may subsequently resort to physical and emotional self-isolation. With support, practice, and time, these individuals may become aware of, develop, and employ strategies for symptom management, including cognitive and behavioral strategies (e.g., mindfulness, self-compassion, emotional regulation, resilience) to cope with challenging symptoms (5).

Importantly, recovery is best promoted through a multi-faceted approach. Individuals living with schizophrenia need psychological and social support stimulated by personally effective wellness strategies and supportive interpersonal relationships. Specifically, *effective* interventions should be flexible in relation to context, functional in approach, strengths-based towards an individual’s potential for growth over impairment, and person-centered to address both body and mind with dignity (6). Among all aspects of recovery, a strong and supportive relationship that fosters emotional warmth and reciprocity with others is the most crucial; as it helps individuals cope with their condition and facilitates healing (7). Additionally, many individuals struggle with adherence to medication regimens due to experiences of, or concerns of potential, side effects, and they should be encouraged to communicate openly about their experiences. Faith and spirituality also serve as valuable coping mechanisms throughout recovery (7).

Moreover, those on the road to recovery are highly motivated by functional recovery, including engagement with educational and vocational pathways (3). For example, in one study, individuals with schizophrenia were found to have a high level of functioning, including holding full-time employment. However, the employment in this example involved manual work, such as lifting and moving things, rather than jobs requiring higher intellectual skills (8). This shows that individuals on the path to recovery, even with conditions like schizophrenia, can achieve meaningful employment and functional recovery, though the job requirements may lie below their actual capacity and work goals.

While medication plays a significant role in managing the symptoms of schizophrenia, *recovery* is a holistic process extending beyond pharmaceutical interventions (9) and is highly influenced by a person’s culture, attitudes, understanding of the recovery process, and access to supporting resources (5). Therefore, the development and provision of recovery services necessitates an awareness of the social determinants of health, including individual and social conceptualizations (i.e., education, values, beliefs, and attitudes) of both schizophrenia and recovery principles.

To date, literature on recovery—either from or in—schizophrenia has been conducted in North America, Europe, and Asia (4, 5, 10), with a dearth of literature representing Ethiopia or sub-Saharan Africa. Similarly, numerous researchers and professionals have pointed out that existing perceptions of recovery are predominantly based on Western socioeconomic and cultural environments, where the *mental health recovery movement* emerged (11–13). Importantly, knowledge or perspectives of recovery may not be transferable to countries with different socio-cultural and economic backgrounds (14). Understanding perspectives on recovery in each context is critical, as a person’s recovery process can be enabled, blocked, or modulated by the cultural and social structure of their environment (15).

To rectify current knowledge gaps, this study focused on exploring recovery experiences in Ethiopian contexts, including the region’s available resources, languages spoken, educational systems, and sociocultural attitudes towards individuals with severe mental illness. Understanding experiences of mental illness recovery is essential to improve the provision and efficacy of healthcare services, as well as sociocultural attitudes toward individuals living with schizophrenia. Thus, this study addresses the following research question: ‘What is the recovery experience of individuals with schizophrenia in Ethiopia?’.

## Methods

### Design

We used Interpretive Phenomenological Analysis (IPA) to explore the recovery experiences of those living with schizophrenia. The interpretive phenomenology approach is aimed at providing a detailed examination of how participants conceptualize their intra- and inter-personal experiences with recovery (16). The IPA approach was used to interpret the meaning of schizophrenia recovery beyond mere clinical description by interpreting participants’ lived experiences. Additionally, the researcher’s prior clinical experiences were used as foundational sources of knowledge to support the study (Holloway & Galvin, 2016); for instance, the first author, Selamawit Kassa (SK), incorporated prior clinical psychiatry and assessment experience as a former intern of the Amanuel Psychiatry Hospital working with individuals diagnosed with schizophrenia. To address the potential bias associated with such prior experiences, coauthors of the present study conducted iterative reviews of the study materials: interview questions, codes, and themes. Further, the first author documented reflexive notes throughout the research process.

### Study setting

This study was conducted at two distinct locations: the University of Gondar Comprehensive Specialized Hospital (UGCSH) in north-western Ethiopia and Felege Hiwot Referral Hospital (FHRH) in Bahir Dar City. UGSCH is a tertiary referral hospital chosen for its broad client base from both urban and rural areas. Typical appointments provided by UGSCH for individuals living with schizophrenia include psychiatric consultation with clients to inquire about their well-being and recurring symptoms, address their questions, and issue prescriptions for medical management of symptoms, the latter of which may provide relief for up to 3 months at a time. The entire process, from consultation to prescription, typically takes no more than 10 minutes, providing efficient delivery of services to individuals seeking assistance.

The second study site of FHRH is situated approximately 500 km from the capital of Ethiopia and 180 km from UGCSH. Both hospitals are situated within the same province and provide relatively similar procedures for individuals living with schizophrenia.

### Sampling and recruitment

The study participants were adults with schizophrenia who were in symptomatic remission, willing to participate, and able to communicate in Amharic language. Participants were recruited through direct communication with professionals who were asked to inform participants about the study. A maximum variation purposeful sampling technique was used to select participants (17). This sampling method was implemented by identifying critical dimensions of population variants, which yielded an emphasis on sampling across urban and rural areas, different gender groups, and ages ranging from 20-60 years. A total of 17 participants were recruited for this study. Before initiating the data collection phase, the principal investigator repeatedly visited both sites to build relationships with psychiatric program staff and to coordinate interviews with participants.

Recruitment continued until the target information power was obtained. The following items were used to decide the obtaining of information power: (1) This study had a narrow research aim, which requires a smaller sample size. (2) To offer adequate information power, a smaller sample size was needed, with participants holding specific characteristics for the study. (3) This study focused on an in-depth analysis of interviews with a few selected participants (18).

The study included participants in active recovery, such that they had been following a medication regimen for at least one year, were not experiencing a relapse in symptoms, and were actively attending outpatient services. Before data collection, the stud’s purpose and possible risks and benefits were explained to all participants to confirm informed consent. All inquiries raised by participants during this process were addressed using lay language to ensure comprehension and clarity regarding their rights as participants. Then, each participant was asked to complete an insight assessment tool (19) to confirm sufficient cognitive capacity for involvement in the interview; of note, schizophrenia may influence individuals’ cognition, necessitating this step. This insight assessment tool was reviewed for face validity by two psychiatrists working at the hospital prior to implementation in this study. The insight assessment tool was comprised of three questions, answered with yes or no responses: 1) do you accept that you have mental illness, 2) do you think that you require treatment, and 3) do you think that you require medication-related to the illness (19)? Only individuals who answered “yes” to questions were selected for participation in the interview phase.

### Data collection

Before commencing data collection, research ethics clearance was obtained from the Queen’s University Health Science and Affiliated Teaching Hospitals Research Ethics Board and the Institutional Review Board of the University of Gondar. Each participant provided written consent to participate in the study. Semi-structured, in-depth interviews were conducted in a quiet meeting room at the hospital for an average of 40 minutes. The interview guide was reviewed by senior researchers for its culture and content appropriateness.

Each interview commenced by gathering sociodemographic information such as age and marital status. Interviews were recorded using a digital recorder, and field notes were made following each session. Data collection took place between October and December 2021. **Table 1** contains the interview protocol used for data collection.

**Table 1:** Interview protocol.

Interview Protocol: Individuals with Schizophrenia	
Questions	Prompts
**Recovery** • What kind of treatment did you receive from the Hospital? • Can you tell me about your recovery experience?	• What is the meaning of recovery for you? • How does it feel to go through the treatments in the Hospital? • What does it feel like when you see improvements/no improvements in your mental health after receiving treatment? • How do you try to move towards recovery other than the hospital treatment?
**Hope and meaning** • What gives you hope in the process of your recovery? • What kind of support do you receive from family and friends?	• What gives you hope in life? • Tell me about the times that you were hopeless, if there were any. • How did you deal with it? • How do you describe the role of spirituality in your recovery?
**Support** • If you have participated in a self-help group, can you tell me what it included and how you found it?	• How much do you feel understood by family and friends? • How does their support make your recovery better? • Can you tell me how the self-help group supported your recovery? • How is the support you receive from the self-help group different from the support from family and friends? • What does it feel like to be supported by a self-help group?
**Culture** • How did the culture (including the society’s norms and religious beliefs) influence your recovery experience?	• How do you deal with the challenges that are related to the culture if there are any? • Can you mention some supportive norms or religious beliefs?

### Trustworthiness

We used different strategies to establish trustworthiness. To ensure conformability, the first author regularly met with the senior researcher (SG) for debriefing sessions. To establish dependability, an audit trail was used, incorporating methodological documentation with a focus on detailed methods and procedures used in the study as well as descriptions of participant and study contexts (20). This process helped to ensure ‘rich description’ of study findings. Further, the first author kept a reflexive journal (21) by documenting her previous knowledge, preconceptions, values, and beliefs about mental illness (especially schizophrenia) and recovery. In addition, coauthors contributed an iterative review of themes, subthemes, respective quotes, and interpretations derived from IPAs of participant interviews.

### Data analysis and interpretation

Data analysis was directed by steps outlined in the Interpretive Phenomenological Analysis protocol (22). Specifically, an inductive data analysis approach was used as no relevant theories or frameworks preexisted in this study, nor were any preexisting categories of analysis used at this stage (23). Participant interviews were first transcribed verbatim and electronically uploaded for use with NVivo 14 analysis software. The first author completed multiple rounds of transcript review for accuracy and completeness. Per the audit trail, procedural and observational notes were taken during the transcript review with the study’s objective in mind. During the second iteration of the transcript review, coding was initiated, which included initial labels of preliminary themes emerging from the captured narratives. Two hundred and eighty initial codes were identified. Preliminary codes were then reviewed, revised, and reframed into groupings (e.g., factors pertaining to obstacles for recovery). This process was repeated, subsequently adding codes to previously identified groups or themes and adding additional themes when needed. From these groupings, themes were established: illness impact, personal coping mechanisms, meanings of recovery, support systems, taking responsibility, etc. After identifying themes within each transcript, connections, similarities, and patterns were identified between transcripts. Final overarching themes were then identified, with supporting themes established as subthemes. These themes were continually subject to ongoing revisions throughout the study.

### Findings

The study included ten female and seven male participants, with a median age of 40 and an interquartile range of 17. Of these participants, seven were female, ten were male, and participants were between 20 and 50 years old. Seven participants were single, six were married, and four were divorced. Eight participants resided in urban areas, while the remaining nine participants resided in rural areas. **Table 2** presents details of each participant’s sociodemographic characteristics.

**Table 2:** Sociodemographic characteristics of participants (n=17).

Name*	Age group	Sex	Marital status	Residence
Agere	20-30	Female	Single	Urban
Alemayehu	30-40	Male	Single	Urban
Alemu	40-50	Male	Single	Urban
Almaw	20-30	Female	Married	Rural
Bekele	20-30	Male	Married	Urban
Etenesh	40-50	Female	Divorced	Rural
Haile	40-50	Male	Divorced	Rural
Meserach	30-40	Female	Single	Urban
Meseret	20-30	Female	Divorced	Urban
Netsanet	40-50	Male	Married	Rural
Tebek	50-60	Male	Married	Rural
Wase	40-50	Male	Single	Urban
Werke	20-30	Female	Married	Rural
Woineshet	20-30	Female	Single	Rural
Worku	40-50	Male	Married	Rural
Yaregal	40-50	Male	Single	Urban
Yitayew	30-40	Male	Divorced	Rural

The names* of participants in the above table are pseudonyms

**Table 3:** Themes and Subthemes.

Themes	Subthemes
Theme I - The meaning of schizophrenia recovery	
Theme II - Barriers to Recovery	• Feeling misunderstood and being limited in social participation • Financial dependency and limited productivity • Hopelessness and suicidal thoughts
Theme III - Facilitators of Recovery	• Family and workplace support • Contributing to family
Theme IV - Coping strategies	• Medication adherence and traditional healers • Spirituality and holy water treatments

The Interpretive data analysis generated four themes and seven subthemes (see **Table 3**).

### Theme I: the meaning of schizophrenia recovery

The analysis revealed that individuals in recovery typically self-identify as ‘recovered’ when they experience reduced symptoms and can engage in meaningful occupations (e.g., employment) and foster interpersonal relationships (e.g., familial connections) or be well enough to enter a supporting roles for others; however, definitions of recovery tended to differ based on the person’s life perspectives and the stage of their diagnosis at the time of inquiry. Notably, all participants focused on ‘recovery from’ mental illness by mentioning symptom relief as a priority:

*“Now I am good. I am not disappointed easily. I go to church. Previously, I used to be disappointed with many things. Sometimes, I even thought about using my gun to kill others. Now, I do not have that kind of problem.” (Tekbek)*

Agere was another participant who often referred to the days before she developed the illness. In the interview, she explained that she could not be productive unless she was free from symptoms:

*“To recover means to live the previous life I used to live. When I recover, I can work and support myself.” (Agere)*

In addition, Meserach explained the ultimate outcome of recovery as her commitment to providing financial support to her family:

*“To recover means, I say that I am recovered when I can help my family. I am supporting my family with many things, which is recovery for me.” (Meserach)*

Other participants often compared themselves to those without the condition. For example, Bekele, encountered numerous challenges in his life and felt he was lagging behind his friends in employment and social milestones. He had perceived recovery as a process of overcoming these restrictions and catching up with his peers across these occupational pursuits:

*“[Recovery means] to be equal to my friends, to go outside and work like my friends. When I see my friends go to work while I am sitting at my door, it makes me sad. I could have been active and lived a normal life if I did not have this illness.” (Bekele)*

Most participants did not accept that their condition was lifelong and hoped for a cure to their illness. Although some were actively employed and economically independent, they felt they needed to be free from symptoms and return to their lives as they were before the onset of their illness. In other words, many of these individuals aimed for, or expected to achieve a “full recovery”, the way people recover from injuries or infections. They mentioned that before having schizophrenia-related symptoms, they had different identities to what they embody presently: happy, productive, independent, and socially involved. They believed that amelioration of their symptoms would equally restore their prior sense of self.

### Theme II: barriers to recovery

The study participants shared many challenges on their road to recovery. As individuals living with schizophrenia, they felt misunderstood by others, with the majority additionally experiencing limited social participation, economic dependency, and hopelessness.

#### Feeling misunderstood and being limited in social participation

Some participants believed that their families and larger communities misunderstood their condition, and as a result, they were limited in their social status and subsequent participation. Participants also highlighted the fluctuating and invisible nature of their symptoms as a primary catalyst for being misjudged. To navigate more challenging days and symptoms, participants generally expressed their need to be able to rely on consistent understanding and support from those around them. Meaningful support was achieved from active listening and empathy from their supports:

*“My neighbors always think that I am not sick. Although I have many symptoms, they still do not believe me. Even my wife and her family say that I am not ill, but the reality is that I am unable to work or be with others due to my illness.” (Yitayew)*

For participants in this study, feeling misunderstood was akin to being invisible, with no one recognizing the depth of their daily struggles. They wanted others to grasp that recovery is a complex, ongoing journey and that fewer symptoms don’t signify an end to their illness:

*“Some of my symptoms have changed, and they believe I am fine. They always compare my current condition with the previous symptoms. Since there are some improvements, they think that I have recovered. No one knows what is happening inside my mind, and they do not know what I experience at night. I always try to tell them, but they do not listen.” (Agere)*

Another challenge faced during recovery was limited social participation. In Ethiopia, regular involvement in social events is a central part of life; therefore missing these gatherings leads to significant feelings of isolation. For example, Etenesh expressed that her ideal social environment would entail a more *co-regulating* sp*ace* where peers could gather and share ideas or experiences more intimately without extraneous or excessive stimuli (e.g., noises, lights, crowds). Her desire for such a space lies in her need to engage more meaningfully with others; however, she noted that these contexts or opportunities were not available as needed:

*“During social gatherings, the noise of people disturbs me very much. When I hear the voices of many people, my ears start to shout. Due to that, I do not go to those social events.” (Etenesh)*

The immense feeling of social isolation was common even in married individuals. Despite their marital status, these individuals faced challenges in fully engaging in social activities or networks outside of their immediate family:

*“My friends invite me to weddings, christenings, or other events, but I do not go because I cannot drink alcohol. They always push me to drink, and I do not drink. Once, I drank alcohol at a friend’s christening ceremony, and then I experienced a relapse and was hospitalized for some time.” (Worku)*

Ultimately, recovery is a long journey that evidently requires conscious and empathetic input from an individual’s supporting relationships. In the minds of participants, misunderstandings and arguments with family members or peers were identified as major obstacles to recovery, and a significant underlying cause of relapses.

#### Financial dependency and limited productivity

Feeling financially dependent was frequently mentioned as a significant source for feelings of inadequacy, low self-esteem, and a perceived lack of control in one’s life. The psychological burden of this lack of self-efficacy often manifested as increased stress, anxiety, and even depression, all of which hindered recovery. Conversely, maintaining a sense of independence and self-sufficiency was a notable protective factor for participants’ mental and emotional well-being, appearing to directly aid in their recovery process.

Participants elaborated on their illness’s direct impact on their productive lives, which resulted in unemployment, economic dependence, and an inability to work and generate income to one’s full or desired capacity. Participants highlighted that they, like anyone else, find meaning in working and being productive, but that they wished for employers to better understand their needs and experiences:

*“My previous working ability is different from my current condition. Previously, I used to be very fast; I did whatever my employers told me to do. Now, I cannot even tolerate the sun. I feel exhausted when I start working.” (Etenesh)*

Periods of financial insecurity, such as unemployment or reduced working hours further impacted individuals’ ability to cover essential expenses. Relying on family support during difficult times is unavoidable and often a crucial safety net for many people. However, it can also place a strain on the individual, their family or support system, and thus the relations between them:

*“I have a shortage of money; currently, I am bothering my family and relatives by asking for money all the time. When I think about this, I become highly stressed.” (Worke)*

Meserach’s story was a powerful example of the transformative impact of regaining financial independence on an individual’s sense of self-worth and well-being. Working as a barber, she was generating income for herself and her family, which brought her a sense of pride and accomplishment. After living with the illness for 8 years, she believed that she knew how to solve conflicts, build meaningful relationships, and cope with stressful days:

*“I always try to help my family. We do not have sufficient income, so I have a barber shop where I work and support my family.” (Meserach)*

The participants’ journey with recovery highlighted the importance of creating opportunities for individuals to regain financial independence.

#### Hopelessness and suicidal thoughts

Stemming from persistent feelings of hopelessness, suicidal ideation was unfortunately common in the sampled population. These feelings were especially exacerbated by struggles to makes progress towards recovery. The illness, especially when it affects one’s ability to work or engage in daily activities, had a profound impact on a person’s mental and emotional well-being – which some participants expressed, led to their suicidal ideations or intents. From the studied sample, three female participants reported feelings of hopelessness after being diagnosed with schizophrenia and two male participants shared their experiences of suicidal ideation.

Participants reported different reasons for feeling hopeless: the loss of emotional and practical support, especially from family members, had a particularly devastating effect on the participants. When individuals lost this support, they felt isolated, abandoned, and overwhelmed. Without adequate support, they felt as though they had nowhere to turn, and that suicide was the only way to escape their pain and suffering. For example, Agere used to have a strong connection with her family. This connection was a significant aspect of her life that gave her a sense of belonging, love, and support. When she lost her family, things changed dramatically. Feeling helpless, exacerbated feelings of loneliness, sadness, and hopelessness, making it even more challenging to cope:

*“Sometimes, when I sit and think, I feel stressed. I become worried about so many things. Especially when I remember my late mother and father, I became very emotional. I used to be highly attached to them, and now I feel hopeless, and I wish I were dead with them.” (Agere)*

Regarding approaches to coping in light of these emotional challenges, Alemu’s story comes to mind. Of the diverse methods of coping recounted by participants, Alemu remarked on the importance of managing his stress through spiritual engagement. Spirituality provided him comfort, hope, and a sense of purpose, offering a source of strength and support during difficult times.

*“Sometimes I thought about committing suicide, and sometimes I talked about it. Usually, I think about suicide when I become depressed, but when I think it is a sin, I change my mind and try to calm down.” (Alemu)*

### Theme III: facilitators of recovery

#### Receiving and providing support is essential for recovery

Individuals with schizophrenia need support from family members and coworkers on their path to recovery. A supportive family can be the difference in an individual living with schizophrenia being able to maintain personal hygiene, have healthy and satisfying food, brainstorm strategies for coping and resiliency, and have someone to look towards for emotional comfort. Support in the workplace was also identified as an important factor in individuals retaining their working status. Considering the challenges of living with a mental illness, understanding, and active support from supervisors was crucial to retaining productivity and promoting progress toward recovery.

#### Family and workplace support

Family members contributed to schizophrenia recovery in different ways. Families provided support for these individuals’ day-to-day activities and needs, especially when the individual had serious symptoms and could not leave home. Specifically, families assisted their loved ones during hospital appointments and by providing meaningful support or resources, such as spiritual services (e.g., access to holy water). Participants emphasized that they might not have received proper and timely diagnosis and treatment if they did not have a family member to advocate for them in their times of need:

*“My sister took me to holy water, and she also brought me to the hospital. Now, I am taking medication, and I am feeling better. She is the only one who understands my situation.” (Woinshet)*

A person with schizophrenia may especially require more attention and support during times of heightened symptoms, where they may be at a greater risk of hurting themselves or others:

*“At first, I used to have serious symptoms. I used to run and walk unconsciously. My family kept their eyes on me and helped me to find treatment.” (Yitayew)*

Most participants expressed their interest in maintaining gainful employment during recovery. However, due to stigma, individuals with mental illnesses like schizophrenia are perceived as less capable of performing activities in the workplace and might be barred from seeking employment or be vulnerable to losing existing jobs. Some participants mentioned that they lost their job as a direct result of their illness, which contributed to their experiences of depression, low self-confidence, and economic dependency.

*“I asked the manager of the organization where I used to work to give me back my job, but he said you are sick and will insult others. I tried to explain that I was feeling better now, but he did not want to listen.” (Meseret)*

In contrast, participants whose supervisors had a better understanding of the condition were able to return to work more easily. For example, Alemu, employed at a government institution in Bahirdar, received support from his supervisors who accommodated his mental health. This support, along with encouragement from his coworkers, motivated and helped him to remain active and productive in his role:

*“The company I am working for pays for my medication expenses. When I take receipts, they reimburse me. They always try to understand my situation.” (Alemu)*

#### Contributing to family

Although schizophrenia affected virtually all areas of their life, most participants continued to play a major role within their families and considered this involvement as an important part of their recovery. Participants were committed to guiding their children, caring for the elderly, and managing daily household activities. For example, Tebek, a 60-year-old farmer, was very dedicated to his family. Trebek felt satisfied and valuable as he had the role of advising and protecting his children. Although there were times when his children didn’t listen to him or follow his advice, he was happy to be involved in their lives as a father:

*“I am leading my family. I guide my children in the right direction. I want them to be disciplined.” (Tebek)*

After losing employment, many participants expressed their attempts to find alternate means of productive occupations, such as increasing their household involvement. For example, despite her symptoms, challenging activities such as collecting firewood and water were manageable for Etenesh. She also could maintain good hygiene and care for her physical needs independently. She believed her life had improved compared to when she was unengaged in her household activities. Staying active and having a role in the household ultimately helped her to stay motivated and engaged in her pursuit of well-being and recovery.

*“Although I feel exhausted, I always try to support my family in the house. I bring wood and water. I also wash our clothes in the river.” (Etenesh)*

### Theme IV: coping strategies

Individuals in recovery described various strategies to cope with their illnesses. Some of the strategies used by the participants of this study included adherence to their medication regimens, going to traditional healers, listening to spiritual songs, praying, and going to holy water sites. Out of 17 participants, 13 mentioned spirituality and holy water treatments as prominent coping strategies. The following subthemes include the details of these coping strategies.

#### Medication adherence and traditional healers

Medication adherence and going to traditional healers were discussed by participants as a major coping strategy. All of the participants in this study stated that they took their medications seriously:

*“Thanks to God, I am taking my medicines properly. I never stopped. Even my family does not remind me. I always remind myself.” (Meserach)*

Participants mentioned that sometimes they had to stop taking their medications due to a shortage of medicine in the pharmacies, the high cost of medication, attempting alternate remedies (e.g., opting instead for holy water sites), or believing that the medication was no longer necessary.

While many participants expressed their voluntary commitment to their medication regimen, other participants appeared less interested in taking their medications consistently. When visiting the hospital, the latter often lacked the opportunity to share their values and perspectives on medication or explore alternative strategies to manage their illness. Simply asking how they were and prescribing medication didn’t seem sufficient or meaningful for their recovery. Taking time to understand their concerns might have made a real difference, per the reflections made by these participants.

#### Holy water treatment, spirituality, and traditional healers

One typical approach participants used for recovery was spirituality. Spirituality was highly practiced due to the belief that mental illness is the result of demon possession. They believed that demons would only be removed through spiritual practices with the hope of complete cure/healing. They wanted to recover from the illness and live their life as they did before the onset of their condition. One common practice was accessing sites with holy water. All participants from this study reported that they had sought out and attended holy water treatments, some once and others multiple times. Some participants solely sought help through holy water sites before opting for help from a hospital or resolved to seek medical help only once their symptoms worsened. Others attended the hospital and holy water treatments simultaneously. For example, Meserach regularly went for holy water treatments:

*“I always go to holy water. My family took me there at first before the hospital. After that, I went to several churches, but side by side, I always take my medication.” (Meserach)*

Some participants tried holy water without taking medications because they believed it had the power to completely heal any illness:

*“I finished my medication while I was in the holy water place. I did not come back to get my medication; I just stayed there. When I started feeling sick, I returned and started retaking it. Then they told me not to stop my medication.” (Alemu)*

Most individuals feared sharing their spiritual practices and especially going to holy water with healthcare professionals as they believed their doctors might not accept them. On the contrary, some individuals were supported by their doctors, which was a significant relief as they were highly dependent on holy water treatment:

*“The doctors told me to go to holy water in addition to my medication. They try to support my interests. Therefore, I am taking my medicines, and I am going to holy water.” (Yitayew)*

Other participants reported that they went to traditional healers to seek treatment. It is usually believed that traditional healers have solutions to many problems. Because mental illness is seen as the result of evil eyes, envy, or demonic possession, people consider traditional healers to have the power to solve mental health problems with their supernatural powers. However, two participants mentioned that they did not find traditional healers helpful.

*“Sometimes I go to traditional healers. I go there for myself, not to hurt others. They gave me traditional medicines, but it did not help me that much.” (Etenensh).*

Regardless of participant’s inclination to seek hospitals or more modernized medical care, most participants attended church activities such as praying, connecting with God, and listening to spiritual songs.

*“When I feel stressed, I listen to spiritual songs, which calms me. Sometimes I sing loudly to myself.” (Alemu)*

## Discussion

This study focuses on the recovery experiences of individuals living in Ethiopia who have been diagnosed with schizophrenia. Participants defined schizophrenia based on their own lived experiences. While all participants focused on symptom reduction and full recovery from illness, some extended their definition to working, supporting family, and being equal with friends. Symptom relief was identified as the greatest concern of individuals with schizophrenia in a recent Indian study (24), while complementary studies indicated that learning, working, and being socially active are essential to recovery (24, 25). All of the participants in this study subscribed to the notion of *recovery from* a mental illness, where they thought they should first recover to continue living a “normal” life (1).

The findings from this study reflected contemporary literature in this area. In summarizing these findings, obstacles to recovery included being misunderstood or stigmatized by others, economic dependency, and hopelessness. Conversely, protective factors were identified by participants as receiving and providing interpersonal support, especially from family and employers. Coping strategies were also highlighted in this study, commonly consisting of visiting traditional healers and holy water sites while continuing medical treatment.

Individuals’ social experiences with schizophrenia were markedly limited in social engagements and punctuated with interpersonal conflicts fueled by others’ misunderstandings of schizophrenia and associated symptomatic behaviors (26, 27). Family members and others living close to those in recovery tend to similarly lack a comprehensive understanding of the nature of mental illness, particularly schizophrenia, and consequently struggle to provide effective support. This compounding lack of understanding within one’s support network and community frequently lead to a dearth of meaningful social connections for individuals in recovery. Feeling misunderstood, affected individuals may then withdraw from social interactions altogether, and initiate or retaliate conflicts with those around them. Previous research has explored the effects of educating family and caregivers about the disorder (28, 29), explaining the source of the illness to the general community (30), and creating support groups (31) to address informational barriers.

Despite barriers to attaining this social and emotional support, participants commonly identified family members’ support as a significant aid in their recovery process. Specific aspects of this support type included providing food, clothing, and assistance in accessing medical services (e.g., accessing holy water sites, and medication reminders). A systematic review also revealed that individuals with schizophrenia often depend on others for basic survival needs such as food, housing, and clothing (7). This indicates that supporting family members with the necessary means for supporting individuals with schizophrenia is crucial.

In addition to receiving social support, participants of this study played an active role in maintaining or working towards their productive occupations, especially in household management or through work in different organizations to support themselves and their family members; further, this notion reflects participants’ efforts to achieve *recovery in* mental illness (32). In essence, ‘recovery in’ mental illness pertains to performance across activities of daily life. Some noted activities included playing with a child, sharing a meal with a friend, listening to music, or chores such as washing dishes. Such interventions help to enhance the person’s sense of pleasure, satisfaction, or self-mastery in daily life (1).

Unsurprisingly, it follows that a person with schizophrenia may experience hopelessness and struggle with suicidal ideation when faced with social and occupational imbalances during recovery. Mostly, the reason for the hopeless and suicidal thoughts comes from losing their loved ones, feeling isolated, conflicts with family members, and economic instability. This result goes in line with a review of the risk factors of suicide among persons who are living with schizophrenia, pointing out that hopelessness, social isolation, hospitalization, recent loss, limited external support, and family instability are significant factors that trigger suicidal acts (33). To mitigate this problem, continuous support should be provided for a person struggling with life circumstances while living with schizophrenia.

Regarding coping strategies, most participants in this study preferred religious practices such as visiting to holy water sites before considering use of hospital services. Others stopped taking their medication when they felt better and continued to pursue healing at holy water treatment sites. The belief that holy water solves every problem was the main reason for choosing religious practices over hospital services. Looking at other studies, a systematic review and meta-analysis also illustrated that faith and spirituality were some of the most significant coping mechanisms used by individuals with schizophrenia (7). Though not notably reported by participants of this study, traditional Chinese spiritual methods (34) and Buddhist teachings (e.g., prayer, meditation, and mindfulness) are coping strategies identified as beneficial for individuals living with schizophrenia (35).

To live a more meaningful and productive life, individuals should understand that their condition is lifelong and, thus, redirect their energy towards effective coping strategies (1, 36). Reflecting this sentiment, mental health advocates echo that people with long-term conditions can work towards reclaiming control of their lives while decreasing the effect of the illness through more informed recovery processes (1). In individuals who had recognized the life-long nature of their condition, many still sought primary treatment at holy water sites, believing that they would be healed. Since being blessed with holy water was the most accepted coping strategy, religious leaders and mental health professionals should discuss how to work together for more optimal provision of care for those living with schizophrenia. Mental health professionals should not discourage the practice but ensure individuals receive medical services alongside holy water modalities. Religious leaders can encourage their followers to go to hospitals and take their medications properly. In addition, professionals can teach religious leaders about mental illness, its causes, treatments, and counseling techniques (37).

Finally, a direction towards bettering individuals’ recovery experiences after the onset of schizophrenia is found—at least in part—in a study conducted in Ethiopia that has explored the effectiveness of recovery-oriented community-based rehabilitation (CBR) interventions. CBR is comprised of psychoeducation, family intervention, support towards maintaining recovery routines, crisis management, returning to work and social activities, and strategies for dealing with stress and stigma. The study findings showed that CBR was effective in decreasing disability in participation and social interaction, symptom severity, and caregiver burden, and showed increased adherence to antipsychotic medication and facility-based care attendance among individuals with schizophrenia (38).

### Implications

This study has implications for mental health practice, policy, and research. *Implications for health practices* include furthering the knowledge base on this issue, which may contribute to organizing more impactful campaigns or programs for increasing awareness in family members, society, and individuals on schizophrenia recovery, especially within rural and semi-urban contexts with unique socioeconomic considerations (39). Building trust and engaging in open conversation with local spiritual leaders and respected community figures is a key first step. After establishing trust, delivering psychoeducation, using a person-centered therapeutic approach, and organizing workshops can be done more effectively. It is essential to respect social norms during these interactions (40).

Ultimately, there is a need for education to emphasize the idea that schizophrenia recovery is a long process with multiple dimensions, or social determinants, underlying health and well-being. Contrary to participants’ common beliefs uncovered in this study, individuals should not wait to be free from symptoms to begin pursuing their meaningful occupations. Professionals should also encourage family members and caregivers to engage individuals with schizophrenia in day-to-day activities that will help them move towards independent living, increase self-confidence, and reduce feelings of hopelessness.

Furthermore, since the illness affects real and perceived self-efficacy and motivation, vocational training should be provided until individuals achieve or regain their capacity and resources to work so that they may become more economically independent. An emphasis on creating client-centered opportunities for economic independence could also help to reduce hopelessness and ensure a smooth recovery process (41).

Moreover, supplementing economic, psychosocial, and informational support with problem-solving, conflict-resolution strategies, and general communication skills (42) may improve participants’ and family members’ collaboration toward individuals’ successful recoveries. Also, participating in self-help groups, group and individual counseling sessions, and social activities may also decrease the rate of suicide ideation and attempts.

*Policy implications:* Policymakers should consider funding vocational training for individuals with schizophrenia and awareness programs for the community and religious leaders. Funding should specifically be provided to family members and individuals for transportation, medication, and other expenses accrued during the recovery process. In addition, policymakers should prioritize integrating patient, family, and community voices into policy development. Moreover, the concept of ‘recovery in’ mental illness should be integrated into policy approaches. This can be done by including the definition of “recovery in mental illness” to policy guidelines or reference materials and promoting strength-based frameworks in policy to progress views of mental illness beyond one’s impairments (i.e., to address the individual holistically, through human-first principles).

*Research implications* include the potential to conduct subsequent studies on topics including the caregiving experiences of family members, the knowledge and attitudes of religious leaders regarding schizophrenia, and experiences of stigma among individuals with schizophrenia.

### Strengths and limitations

This study’s strengths are owed in part to it being the first study in Ethiopia to interview individuals who are actively engaged in schizophrenia recovery; importantly, this study aims to set a precedent of client-centered research, raising these participants’ voices towards their perspectives, contributions to a more fulsome community, and their need to achieve that vision. Contemporary studies have predominantly focused on the voices of professionals or family members to understand the experience of a person with serious mental illness; however, filling the gap more directly with the voice of those represented by this research is the best way to promote awareness. Moreover, using an IPA is an appropriate way to achieve a more in-depth understanding of the experience of people in recovery. Finally, the first author is from Ethiopia, which lends a more culturally informed lens to this study (e.g., language, geography, cultural beliefs, and values).

Limitations of this study include recruitment sources and recruitment limits based on self-reported diagnoses. In the first limitation, participants were selected from hospitals, rendering the results less representative of individuals who have not received services, such as those seeking solely spiritual support modalities. However, individuals who had not received treatment were ineligible for this study due to its specific focus. Including people not receiving hospital care would have required community-based data collection, which was not feasible as the study’s data collection period coincided with the COVID-19 outbreak. The second limitation is that the assessment tool used to identify participants had the potential of leaving out participants who did not identify with or acknowledge their mental illness, and those who did not take medications. However, those who believed that they did not have mental illness were not the intended targets of this study. Additionally, although the insight assessment tool was previously used Nigeria, Africa (19) it was not previously used nor validated for in Ethiopia.

## Conclusion

This study aimed to explore the recovery experiences of persons with schizophrenia in northwestern Ethiopia. The result of this study showed that individuals with schizophrenia face various challenges during the process of recovery and use different social, productive, medical, and spiritual strategies to cope. The concept of ‘recovery from’ mental illness was highly evident among participants of this study, and this indicates that ‘recovery in’ mental illness should be introduced to persons who are living with schizophrenia and family members. Generally, it is recommended that professionals from the Ministry of Health, mental health professionals, and researchers organize awareness programs for traditional healers, religious leaders, employers, and the community.

## Data Availability

The raw data supporting the conclusions of this article will be made available by the authors, without undue reservation.
